# Complete mitochondrial genome of *Chrysolophus pictus* (Galliformes: Phasianidae), a national protected wild pheasant in China

**DOI:** 10.1080/23802359.2020.1722762

**Published:** 2020-02-03

**Authors:** Wei Bai, Jun Zhu, Zhu-mei Ren

**Affiliations:** a School of Life Science, Shanxi University, Taiyuan, China;; b Biodiversity Research Center of Shanxi, Taiyuan, China

**Keywords:** *Chrysolophus pictus*, Phasianidae, Mitochondrial genome, Phylogeny

## Abstract

The complete mitochondrial genome (mitogenome) of golden pheasant *Chrysolophus pictus* from North China was sequenced by the shotgun genome skimming methods. The mitogenome of *C. pictus* was 16,678 bp in length, consisting of 13 protein-coding genes, 22 tRNA genes, two rRNA genes and one non-coding control region (D-loop). Its overall base composition was 30.4% A, 24.8% T, 31.2% C and 13.6% G. All protein-coding genes had a typical ATG initiation codon except *COX1* with GTG and terminated with a TAN codon, whereas *COX1* terminated with a codon of AGG, *COX3, ND2* and *ND4* terminated with a single T. The ML phylogenetic tree constructed using 13 protein-coding genes showed that *Chrysolophus* species formed a monophyletic group, which was sister to the clade clustered by the two genera *Crossoptilon* and *Lophura*.

The golden pheasant or Chinese pheasant *Chrysolophus pictus* is a gamebird of the order Galliformes (Gallinaceous birds) and the family Phasianidae (Pheasants) (Jobling 2010). The species is endemic in China, and its core distribution area is at Gansu and Qinling area (Zhao 2001). The National Forestry Administration of China listed the species into ‘Red Book of Endangered Animals of China’ as the protected wild animal level: Level 2 and also listed in IUCN Red List of Threatened Species ver. 3.1 regarded as ‘Least Concern’ (2012). The previous studies on *C. pictus* mainly focused on possible evolutionary significance of egg-white proteins, egg production performance and nutritional value (Baker 1965; Gao et al. 2016; Kullu et al. 2017). Genetic characteristics are very important in species conservation, however, there is no more reports on the mitogenome of the golden-pheasant *C. pictus* in North China. We here performed the complete mitogenome sequencing of *C. pictus* for the purpose of better understanding the pheasant genomic structure, function and genetic variation.

The feather material from a healthy adult individual of *Chrysolophus pictus* by undamaged method in Manghe National Nature Reserve (112°22′10″E, 35°12′30″N, altitude 310 m), Shanxi, China, and the total genomic DNA (Voucher No. Ren-Z17) was stored at the Zoology Herbarium of School of Life Science in Shanxi University. The mitogenomic sequence was obtained using the shotgun genome skimming method on an Illumina HiSeq4000 platform (Zimmer and Wen 2015), and was then de novo assembled by SPAdes v. 3.7.1 (Bankevich et al. 2012).

The complete mitochondrial genome of *Chrysolophus pictus* is 16,678 bp in length (GenBank Accession No. MN857545), and consists of 30.4% A, 24.8% T, 31.2% C and 13.6% G with a little higher A + T content (55.2%) than that of G + C (44.8%), which is basically consistent with those of other Phasianidae species. The complete mitogenome of *C. pictus* totally included 13 protein-coding genes (*ND1-6*, *ND4L*; *COX1-3*; *ATP6* and *ATP8*; and *Cyt B*), 22 tRNA genes, two rRNA genes (12S and 16S rRNA) and one non-coding control region (D-loop). All the protein-coding genes had a typical ATG initiation codon except *COX1* with GTG. Most genes terminated with TAA codons except *ND6* and *Cyt B* had TAG termination codons, and *COX1* with AGG codon, whereas, *COX3*, *ND2* and *ND4* each terminated with a single T. The genetic variation among the four *Chrysolophus pictus* individuals is much lower with average similarity 99.6%.

We downloaded the complete mitogenomic sequences of Phasianidae species from GenBank with three Odontophoridae species as outgroups to construct the phylogenetic relationship of *Chrysolophus pictus* and other Phasianidae species in RAxML program under the GTRGAMMA model with 1000 bootstrap replicates (Stamatakis 2014). The phylogenetic analyses indicated that *Chrysolophus* is sister to *Phasianus* but with poor support, and they are close to the clade clustered by the two genera *Crossoptilon* and *Lophura* (Figure 1). In conclusion, our study contributes to understand the structure and composition of the complete mitogenome of *C. pictus* and the evolutionary history and genetic resource conservation of Phasianidae.

**Figure 1.**
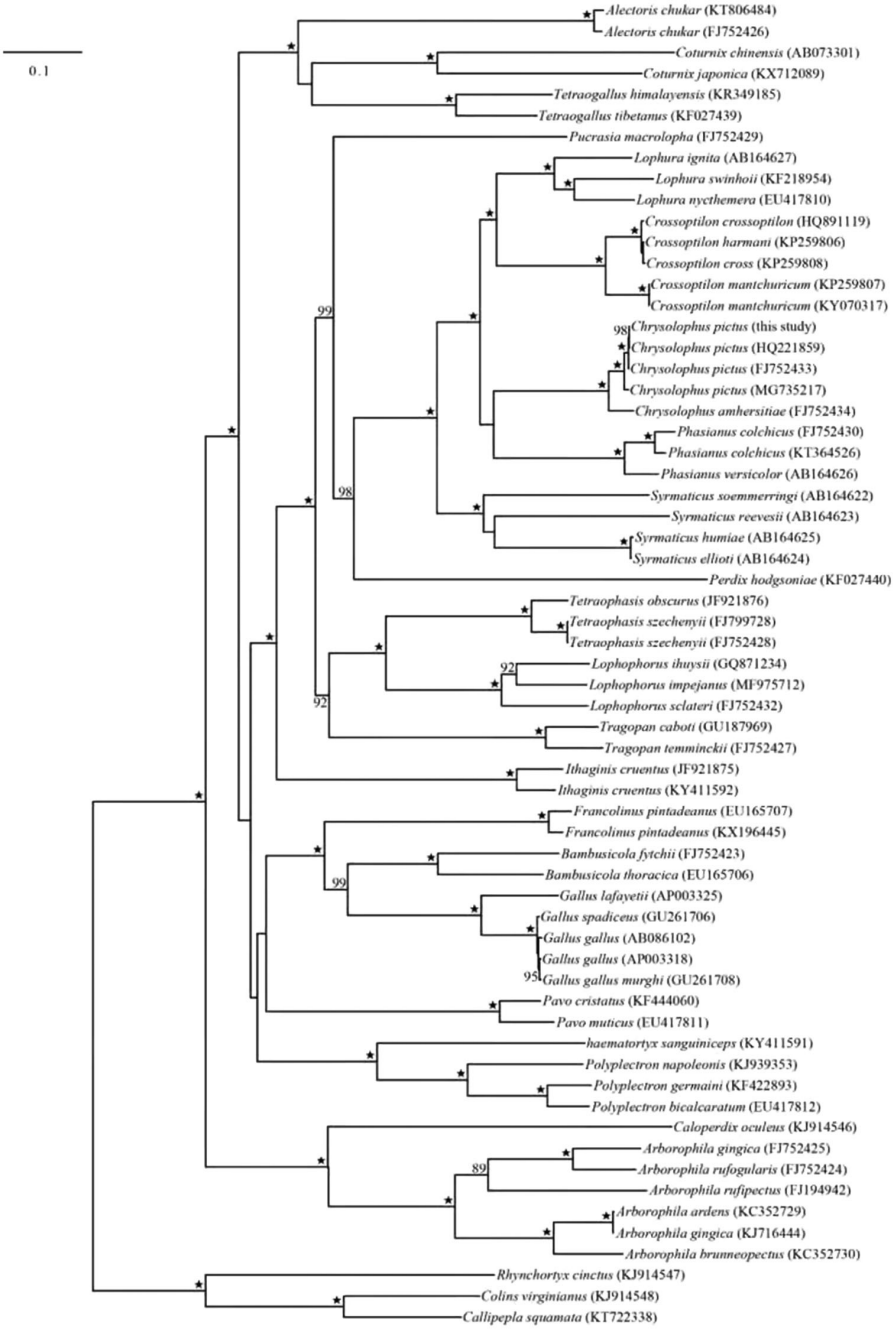
Maximum likelihood phylogenetic tree of *Chrysolophus pictus* and other Phasianidae species based on 13 protein-coding genes. Numbers associated with branches are BS > 75, and “★” represents nodes with 100% BS.

## References

[CIT0001] Baker CM. 1965. Molecular genetics of avian proteins IV. The egg-white proteins of the Golden pheasant, *Chrysolophus pictus* L and Lady Amherst’s pheasant, C. Amherstiae Leadbeater, and their possible evolutionary significance. Comp Biochem Physiol. 16:93–101. 5861537 10.1016/0010-406x(65)90166-0

[CIT0002] Bankevich A, Nurk S, Antipov D, Gurevich AA, Dvorkin M, Kulikov AS, Lesin VM, Nikolenko SI, Pham S, Prjibelski AD, et al. 2012. SPAdes: a new genome assembly algorithm and its applications to single-cell sequencing. J Comput Biol. 19(5):455–477. 22506599 10.1089/cmb.2012.0021 PMC3342519

[CIT0003] Gao GQ, Song LS, Tong B, Li GP. 2016. Expression levels of GSTA2 and APOD genes might be associated with carotenoid coloration in golden pheasant (*Chrysolophus pictus*) plumage. Zool Res. 37(3):144–150. 10.13918/j.issn.2095-8137.2016.3.144 PMC4914577 27265652

[CIT0004] Jobling JA. 2010. The Helm Dictionary of Scientific Bird Names. London: Christopher Helm. p. 105, 306.

[CIT0005] Kullu SS, Das A, Bajpai SK, Garg AK, Yogi RK, Saini M, Sharma AK. 2017. Egg production performance, egg yolk antioxidant profile and excreta concentration of corticosterone in golden Pheasants (*Chrysolophus pictus*) fed diets containing different levels of green vegetables. J Anim Physiol Anim Nutr. 101(5):e31–e42. 10.1111/jpn.12555 27862403

[CIT0006] Stamatakis A. 2014. RAxML version 8: a tool for phylogenetic analysis and post-analysis of large phylogenies. Bioinformatics. 30(9):1312–1313. 24451623 10.1093/bioinformatics/btu033 PMC3998144

[CIT0007] Zhao ZJ. 2001. Avifauna of China (no Passeriformes). China: Jilin Science and Technology Press; p. 401–402.

[CIT0008] Zimmer EA, Wen J. 2015. Using nuclear gene data for plant phylo-genetics: progress and prospects II. Next-gen approaches. J Syst Evol. 53(5):371–379.

